# Advances in targeted therapy mainly based on signal pathways for nasopharyngeal carcinoma

**DOI:** 10.1038/s41392-020-00340-2

**Published:** 2020-10-23

**Authors:** Yuanbo Kang, Weihan He, Jincheng Qiao, Qiuyong Guo, Caiping Ren, Jingyu Hu, Hongjuan Xu, Xingjun Jiang, Lei Wang

**Affiliations:** 1 Department of Neurosurgery, Cancer Research Institute, Xiangya Hospital, Central South University, 410008 Changsha, Hunan China; 2 Cancer Research Institute, Collaborative Innovation Center for Cancer Medicine, School of Basic Medical Science, Central South University, 410008 Changsha, Hunan China; 3 The NHC Key Laboratory of Carcinogenesis and The Key Laboratory of Carcinogenesis and Cancer Invasion of the Chinese Ministry of Education, Xiangya Hospital, Central South University, 410008 Changsha, Hunan China

**Keywords:** Cancer, Oncology

## Abstract

Nasopharyngeal carcinoma (NPC) is a malignant epithelial carcinoma of the head and neck region which mainly distributes in southern China and Southeast Asia and has a crucial association with the Epstein–Barr virus. Based on epidemiological data, both incidence and mortality of NPC have significantly declined in recent decades grounded on the improvement of living standard and medical level in an endemic region, in particular, with the clinical use of individualized chemotherapy and intensity-modulated radiotherapy (IMRT) which profoundly contributes to the cure rate of NPC patients. To tackle the challenges including local recurrence and distant metastasis in the current NPC treatment, we discussed the implication of using targeted therapy against critical molecules in various signal pathways, and how they synergize with chemoradiotherapy in the NPC treatment. Combination treatment including targeted therapy and IMRT or concurrent chemoradiotherapy is presumably to be future options, which may reduce radiation or chemotherapy toxicities and open new avenues for the improvement of the expected functional outcome for patients with advanced NPC.

## Introduction

Nasopharyngeal carcinoma (NPC) is a malignant tumor derived from head and neck epithelial cells. In 2018, there were 129,000 new cases of NPC worldwide, accounting for 0.7% of global cancers, of which the mortality rate of male patients was three times than of females.^1^ The majority of NPC patients are geographically localized to Southeast Asia, while China is one of the countries that have the highest incidence and mortality rates.^2^ According to the World Health Organization, NPC is classified into various histological subtypes: keratinizing squamous cell carcinoma (KSCC), non-keratinizing differentiated carcinoma (NKDC), and non-keratinizing undifferentiated carcinoma (NKUC).^3^ In fact, non-keratinizing subtype closely associated with Epstein–Barr virus (EBV) infection comprises up to 95% of cases in endemic areas, such as southern China, while squamous cell cancers are common in the United States and Europe.^4,5^ In addition, patients with recurrence or metastasis often have a very poor prognosis due to distant metastasis.^6,7^

The genetic and epigenetic alterations of NPC have been unveiled by the constant genome-wide studies, which involve cytogenic, allelotyping, CGH, and array-based CGH analysis.^8–11^ Not only copy numbers losses of chromosome 1p, 3p, 9p, 9q, 11q, 13q, 14q, and 16q but also amplification of chromosome 1q, 3q, 8q, 12p, and 12q were detected in primary NPC by CGH.^12–15^ Promoter hypermethylation of RASSF1A at 3p21.3, homozygous deletions of CDKN2A at 9p21.3 and TGFBR2 at 3p24 have been regarded as triggers of tumorigenesis.^10,16,17^ The alterations of the somatic genome were reported when applying NGS technology on normal and tumor specimens, most of which are either EBV-positive or non-keratinizing NPC.^18^ As an important cause of NPC, EBV infection leads to the expression of various latent viral proteins, such as latent membrane proteins (LMP1, LMP2), BamH1-A fragment rightward reading frame 1 (BARF1) and nuclear antigen (EBNA1).^19–21^ At the same time, EBV infection also leads to the accumulation of a set of non-coding RNAs.^22^ Recently, Hong et al.^23^ found that circCRIM, a kind of circRNAs, promote NPC metastasis and epithelial–mesenchymal transition (EMT) through binding miR-422a to cancel its inhibition to FOXQ1. These viral and host’s genomic products affect the signal transductions and cellular mechanisms of normal nasopharyngeal epithelial cells and have major roles in the pathogenesis of NPC.^24^ Mutations and repair disorders caused by viral infection are important causes of malignant changes in the nasopharyngeal epithelium. As a main substitution type at NpCpG trinucleotides, the C>T transitions results of spontaneous deamination of 5-methylcytosine, followed by a corrupted DNA mismatch repair signature,^25^ while C>G and C>T mutations at TpCpN trinucleotides are linked to catalytic polypeptide-like-mediated signature and the apolipoprotein B mRNA-editing enzyme.^26^ What’s more, there are mismatch repair (MMR) gene mutations and a primary NPC subgroup with a hypermutation phenotype.^25^ C666-1 which is EBV-positive has its own characteristics of inactivating PMS2 mutation and hypermutation phenotype.

The epigenetic machinery of NPC cells could be altered to reprogram the epigenomes of virus and host cells through EBV-encoded proteins. LMP1 contributes to the expression of DNA methyltransferase and the interaction of EBNA3A and EBNA3C with co-repressor of transcription CtBP could modulate polycomb group protein, which could form higher-order chromatin structures to silence target genes.^27,28^ The promoter hypermethylation of RASSF1A, BLU, CDKN2A, and DLEC1 could be detected in NPC, which have roles in DNA damage response, stress response, cell proliferation during G1 and STAT3 signal pathway.^29–31^ What’s more, a variety of tumor-related genes are epigenetically modified, involving transcription factors, enzymes, mitotic checkpoint regulators, cadherins, non-coding RNAs. As the most significantly hypermethylated gene, HOPX is highly associated with early distant metastasis in NPC.^32^ The antiangiogenic effect and anti-cancer activity of metalloprotease (MMP)-19 are inhibited in NPC by allelic deletion and promoter hypermethylation.^33^ As a mitotic checkpoint regulator, promoter hypermethylation of CHFR could cancel the impediment to chromatin condensation.^34^ Sun et al.^35^ found aberrant methylation of CDH13 could be detected in 89.7% primary NPC tumors with methylation-specific PCR. Long non-coding RNA MEG3 is also silenced epigenetically, which can inhibit proliferation, colony formation, induce cell cycle arrest, and has tumor-suppressive properties in vivo and in vitro.^36^ Leong also found abnormal histone bivalent switch is linked to suppressing DNA damage repair gene, which indicates another kind of epigenetic modification.

Targeted therapy involves the design of specific drugs that bind specifically to oncogenic targets within tumor cells to inhibit the development of tumors. MicroRNAs inhibit transcription and translation by binding to the 3′-UTR of target mRNA and affect the expression of target proteins, which has an important role in the genesis and development of tumors.^37,38^ In addition, abnormal activation and silencing of signal pathways in tumor cells also have a crucial role in tumor activities.^39–42^ Imbalance of the phosphatidylinositol 3-kinase (PI3K)/Akt/mTOR signal pathway is associated with malignant transformation and apoptosis of tumor cells, and metastasis and radioresistance of tumor tissues,^43^ while abnormal activation of the VEGF pathway is associated with angiogenesis in tumor tissue.^44^ Upregulation of the Wnt/β-catenin pathway in tumors is closely related to radioresistance,^45^ activation of the Notch pathway is widely present in human tumors by regulating self-renewal of cells with inhibition of differentiation.^46,47^ Abnormal activation of the Mitogen-activated protein kinase (MAPK) pathway is associated with proliferation, migration, invasion, and angiogenesis of tumor cells.^48,49^

In recent years, NPC patients receiving chemoradiotherapy have a poor quality of life, along with severe side effects such as bone suppression.^50^ However, targeted therapy can accurately identify and treat NPC cells with low toxic and side effects, suggesting a broad prospect of targeted therapy in the clinical treatment of NPC.^51^ In this article, we reviewed crucial molecules in signal pathways and miRNAs/lncRNAs in NPC cells studied in recent five years, regarding their roles in the promotion or suppression of NPC and functions as potential therapeutic targets of this disease. In addition, we present future perspectives of biomarker-based treatments and clinical diagnoses in NPC.

## Crucial signal pathways related to targeted therapy of NPC

Aberrant activation of signal pathways brings about a variety of human diseases. Abnormal transmembrane signal pathways, including prosurvival pathways (PI3K/Akt, NF-κB, MAPK, STAT3, Wnt/β-catenin) and proapoptosis pathways (p53, endoplasmic reticulum stress) in NPC cells, have been proved to be associated with the development, progression, and prognosis of NPC by influencing biological processes such as cell cycle, apoptosis, and DNA repair. They are of potential clinical significance for personalized treatment strategies for NPC. The order of descriptions below corresponds to the depth of the last five years of research.

### PI3K/Akt pathway

PI3K phosphorylates phosphatidylinositol 4,5-bisphosphate (PIP2) and converts it to phosphatidylinositol 3,4,5-trisphosphate (PIP3), which is critical for activation of Akt.^52^ Phosphatase and tensin homolog (PTEN) is a phosphatase that dephosphorylates PIP3, and dephosphorylation of PIP3 can block PI3K/Akt signal pathway.^53^ mTOR is a serine/threonine kinase that one of the upstream regulators of mTOR is the PI3K/Akt signal pathway.^54^ In the PI3K/Akt pathway, infrequent mutations of activators or regulators (PIK3CA, PTEN, PIK3R1, AKT2, and mTOR) were observed.^25^ The 110α catalytic subunit of PI3K is encoded by the PIK3CA gene and PIK3CA amplification likely results in the activation of the PI3K/Akt pathway.^55,56^ The activation of the PI3K/Akt pathway is also related to EBV-encoded latent membrane proteins 1, 2A, 2B.^57^

NPC is highly associated with EBV infection and the major oncogenic proteins of EBV are LMP1 and LMP2A.^58,59^ LMP1 induces anti-TNF-related apoptosis-inducing ligand (TRAIL) activity in NPC cells by activating the PI3K/Akt pathway, thereby promoting the progression of NPC.^60^ DNA methyltransferase 1 (DNMT1) can mediate the downregulation of PTEN by LMP1 thereby activating Akt signal.^61^ Downregulation of DNMT1 can reduce the methylation level of the miR-152 gene and improve the expression of miR-152, leading to lower expression of DNMT1 mRNA and protein which inhibits the migration and invasion of tumor cells.^62^ On the other hand, Li et al.^63^ found that DNMT1 can promote EMT and metastasis of NPC by inhibiting the miR-142-3p/Zinc-finger E-box binding homeobox 2 (ZEB2) axis. LMP1 can induce lipid synthesis mediated by sterol regulatory element-binding protein 1 (SREBP1) through the mTOR signal pathway to promote cell proliferation and tumor invasion.^64^ LMP2A-mediated activation of the PI3K/Akt/mTOR/HIF-1α signal cascade can lead to vasculogenic mimicry (VM).^65^

Recent studies provide a basis for the selection of potential targets for targeted therapy of NPC.

Sodium butyrate (NaBu) may inhibit Akt/mTOR axis activity by promoting the degradation of EGFR in histone deacetylase 6 dependent way in NPC cells.^66^ Mitogen-activated protein kinase-activated protein kinase 2 (MK2) is a serine/threonine kinase.^67^ MiR-296-3p can block MK2-induced PI3K/Akt/c-Myc signal pathway by directly targeting and downregulating the expression of MK2 thereby inhibiting cell cycle and EMT as well as cisplatin resistance.^68^ Studies have demonstrated that miR-374a inhibits PI3K/Akt signal pathway, cell cycle, and EMT signal by directly targeting and downregulating Cyclin D1(CCND1), while CCND1 can activate the PI3K/Akt pathway to increase the expression of c-Jun to downregulate miR-374a.^69^ As one of the most upregulated lncRNAs, FAM225A could enhance the expression of integrin β3 (ITGB3) by functioning as a miR-590-3p and miR-1275 sponge, thus activating FAK/PI3K/Akt signal pathway related to proliferation and invasion in NPC.^70^ FOXO1 can inhibit the expression of myosin heavy chain 9 (MYH9) by inhibiting the PI3K/Akt/c-Myc pathway and activating p53/miR-133a-3p axis thereby inhibiting the stem cell characteristics, metastasizing and enhancing the sensitivity to cisplatin of NPC cells.^71^ MiR-9 inhibits the activation of the PI3K/Akt pathway by targeting and downregulating midkine (MDK).^72,73^ MiR-92a promotes NPC cell migration and invasion by targeting and downregulating PTEN causing the activation of the PI3K/Akt pathway.^74^ EBV-miR-BART7-3p, an EBV-encoded BART-microRNA highly expressed in NPC, activates the PI3K/Akt signal pathway, induces the expression of c-Myc and c-Jun, and promotes the growth, proliferation, and tumorigenesis of NPC cells.^75^ For the downregulation of fibroblast growth factor receptor 2 (FGFR2) can enhance the activation of the caspase pathway caused by cisplatin.^76^ Fibroblast growth factor 2 (FGF2) is the upstream molecule of the PI3K/Akt signal pathway so that FGF2/FGFR2 has become a crucial target in the targeted therapy of NPC as well.^77^ MiR-16 can directly target and inhibit FGF2, resulting in the inhibition of both PI3K/Akt and MAPK signal pathways, causing the inhibition of proliferation, migration, and invasion of NPC cells.^77^ Collagen type I alpha 1 (COL1A1) can regulate the radioresistance of tumor cells through the PI3K/Akt pathway.^78^ MiR-29a can induce radiosensitivity in NPC cells by directly targeting and downregulating COL1A1.^79^ RNA-binding motif protein 3 (RBM3) inhibits cancer cells apoptosis by activating the PI3K/Akt/Bcl-2 signal pathway thereby enhancing radiation tolerance.^80^ Ionizing radiation (IR) can increase the expression of phosphatase 1 nuclear-targeting subunit (PNUTS) in NPC cells, which induces EMT by activating the PI3K/Akt signal pathway.^81^ Annexin A1 inhibits the autophagy pathway of NPC cells by increasing Akt phosphorylation level and membrane transport to activate the PI3K/Akt pathway thereby upregulating Sequestosome-1 (SQSTM1) and Snail, inducing EMT in NPC cells and promoting NPC cell migration, invasion, and metastasis.^82^ Besides, IL-8 can regulate NPC metastasis by means of activating Akt signaling and inducing EMT of NPC cells.^83,84^ Downregulation of cyclin-dependent kinase inhibitor 3 (CDKN3) decreases the phosphorylation of Akt and inhibits the increase in the size and weight of transplanted tumors.^85^ C-Src in NPC cells promotes the EMT process to improve the metastatic ability of cancer cells by activating the PI3K/Akt pathway.^86^ The cell membrane alteration also makes an important impact in metastasis. Flotillin-2 (Flot-2), a key component of lipid rafts, is found to be a high sensitivity biomarker for lymph node metastasis in NPC.^87^ Silencing Flot-2 expression in 5-8F cells suppressed metastasis and proliferation as a result of inhibiting NF-κB and PI3K/Akt3 signal pathways.^88^ Besides, the important role of Flot-2 in the progression of NPC might be partially linked to its interaction with PLC-δ3 (PLCD3).^89^ In addition, as a type I transmembrane glycoprotein, the role of epithelial cell adhesion molecule (EpCAM) in different cancers is distinct which might depend on the cell type and microenvironment. In head and neck squamous cell carcinomas (HNSCCs), the extracellular domain of EpCAM (EpEX) has a role of a ligand of EGFR that induces EGFR-dependent proliferation but counteracts EGF-induced EMT.^90^ On the contrary, the overexpression of EpCAM could promote EMT and stemness and metastasis through PTEN/AKT/mTOR signal pathway in NPC cells.^91^ Li et al. found that EphA2 is associated with the formation of NPC as a tumor promoter. In particular, the phosphorylation of EphA2 at serine 897 site act as a predominant role in the clinical metastasis of NPC. pS897 EphA2 activates PI3K through the regulatory subunit p85 of PI3K, which may promote NPC invasion, metastasis, and cancer stem cell characteristics.^92^

Leucine zipper tumor suppressor 2 (LZTS2) can inhibit the activation of PI3K/Akt signal pathway causing the inhibition of tumorigenesis and radioresistance in NPC.^93^ LMP1 can increase miR-155 expression and miR-155–UBQLN1 axis can affect the proliferation, cell cycle, and EMT progression of NPC cells by activating PI3K/Akt pathway.^94^ MiR-18a can activate mTOR by targeting and downregulating suppressor of morphogenesis in genitalia 1 (SMG1), which is an antagonist of mTOR in NPC. Nuclear factor-kappa B (NF-κB) activation and LMP1 expression can induce miR-18a expression in NPC cells.^95^

In summary, in addition to directly targeted inhibition of PI3K, Akt, and mTOR, targeted inhibition of HIF-1α, MK2, CCND1, lncRNA FAM225A, MYH9, MDK, miR-92a, miR-18a, EBV-miR-BART7-3p, FGF2, COL1A1, RBM3, PNUTS, Annexin A1, IL-8, CDKN3, c-Src, Flot-2, EpCAM, OCT4, BEX3, and targeted activation of LZTS2, UBQLN1, SMG1 may become potential therapeutic strategies that affecting the PI3K/Akt pathway in NPC.

### p53 pathway

TP53 was consistently regarded as one of the significantly mutated genes in NPC through whole-exome sequencing (WES)/whole-genome sequencing studies (WGS).^26^ As a tumor suppressor gene, its mutation is related to cancer progression and treatment resistance.^96^ The level of p53 protein in cells is tightly controlled by its negative regulator, the E3 ubiquitin-protein ligase murine double minute 2 (MDM2).^97^ Nutlin-3 competitively inhibits MDM2 and activates the p53 pathway, thereby increasing the sensitivity of NPC cells to cisplatin.^98^ MDM2 inhibitor RG7388 can activate the p53 pathway, increase the expression of p21 protein and caspase family protein that mediate cell cycle arrest, ultimately leading to cell cycle arrest, cell proliferation inhibition, and apoptosis induction.^99^

Other recent studies have provided evidence for the selection of p53-related targets to NPC treatment. Shi et al. found that cycloxygenase-2 (COX-2) may suppress chemotherapy-induced senescence by inhibiting the activation of p53. This study provided an experimental basis for anti-tumor therapy by targeting COX-2.^100^ FOXO1 suppresses the expression of MYH9 by inhibiting the PI3K/Akt/c-Myc/p53/miR-133a-3p pathway, thereby inhibiting the stem cell characteristics, metastasis and increasing cisplatin sensitivity of NPC cells.^71^ B-cell-specific Moloney murine leukemia virus insertion site 1 (Bmi-1) can improve the proliferation, migration, and invasion ability of NPC cells by inhibiting the p16^INK4a^-p14^ARF^-p53 pathway. Targeted silencing of Bmi-1 may have potential clinical significance in the treatment of NPC.^101^ As a tumor suppressor gene, Pin2 telomeric repeat factor 1-interacting telomerase inhibitor 1 (PinX1) inhibits cell proliferation, migration, and invasion by regulating p53/miR-200b-mediated transcriptional inhibition of Snail1, Twist1, and Zeb1, thereby inhibiting EMT of CD133(+) cancer stem cells (CSCs) in NPC.^102^ Furthermore, Zhao et al.^103^ found that cisplatin treatment can induce the upregulation of miR-125a and miR-125b expression, and these miRNAs can target p53 mRNA and reduce p53 protein-induced apoptosis, which may be one of the causes of cisplatin resistance in NPC cells.

For the p53 pathway, targeted inhibition of MDM2, COX-2, MYH9, Bmi-1, miR-125a, and miR-125b may become potential therapeutic strategies in NPC.

**Fig. 1:**
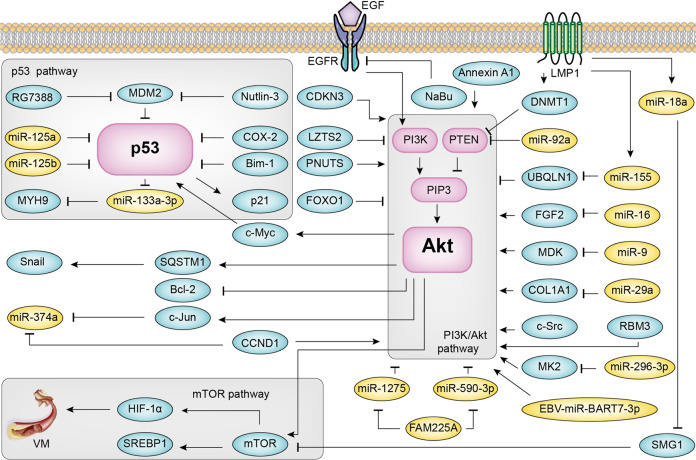
PI3K/Akt signal pathway, p53 signal pathway, and mTOR signal pathway in NPC. LMP1 latent membrane protein 1, LMP2A latent membrane protein 2A, PIP2 phosphatidylinositol 4,5-bisphosphate, PIP3 phosphatidylinositol 3,4,5-trisphosphate, PTEN phosphatase, and tensin homolog, PI3K phosphoinositide 3-kinase, DNMT1 DNA methyltransferase 1, SREBP1 sterol regulatory element-binding protein 1, VM vasculogenic mimicry, NaBu sodium butyrate, MK2 mitogen-activated protein kinase-activated protein kinase 2, CCND1 cyclin D1, MYH9 myosin heavy chain 9, ITGB3 integrin β3, MDK midkine, FGF2 fibroblast growth factor 2, COL1A1 collagen type I alpha 1, RBM3 RNA-binding motif protein 3, PNUTS phosphatase 1 nuclear-targeting subunit, CDKN3 cyclin-dependent kinase inhibitor 3, SQSTM1 Sequestosome-1, LZTS2 leucine zipper tumor suppressor 2, UBQLN1 ubiquilin1, SMG1 suppressor of morphogenesis in genitalia 1, MDM2 murine double minute 2, COX-2 cycloxygenase-2, Bmi-1 B-cell-specific Moloney murine leukemia virus insertion site 1, EMT epithelial–mesenchymal transformation. Among them, LMP1, mTOR, PI3K, HIF-1α, miR-92a, Annexin A1, CDKN3, OCT4, c-Src, and COX-2 can be used as prognostic markers of NPC. The preclinical studies of NPC show that HIF-1α, COX-2 has a good research value in targeted therapy. EGFR inhibitor, VEGF inhibitor, VEGFR inhibitor, and Akt inhibitor show positive effects and have less and tolerable adverse reactions in phase II clinical trials of NPC

A summary of the PI3K/Akt signal pathway, p53 signal pathway, and mTOR signal pathway in NPC is shown in Fig. 1.

### NF-κB pathway

NF-κB is a key multipotent dimer transcription factor.^104^ Studies have shown that the activation of the NF-κB pathway is closely related to persistent infection of EBV, the formation of immortal nasopharyngeal epithelial cells and immunosuppressive environment, cancer stem cells generation, and metabolic reprogramming.^105^ Through the WES, several NF-κB signaling negative regulators NFKBIA, CYLD, and TNFAIP3 were found multiple loss-of-function mutations. The loss-of-function mutations in NFKBIA lead to protein truncation and result in the altered NF-κB activity, which contributes to NPC tumorigenesis.^26^

LMP1 is believed to activate the NF-κB signal pathway.^106^ In EBV-associated NPC cells, LMP1-activated NF-κB binds to the promoter region of the miR-203 gene and inhibits miR-203 expression, Cadherin 6 (CDH6) as a direct target of miR-203 upregulates in turn induced EMT and promotes metastasis. Furthermore, EBV-negative NPC cells can induce the EMT potential through the uptake of exo-LMP1.^107^ The NF-κB inhibitor aspirin can reverse EMT, reduce the release of exo-LMP1, promote the expression of miR-203, and inhibit the metastasis of NPC cells in nude mice.^108^ Provirus integration site for Moloney murine leukemia virus 1 (Pim1) is a serine/threonine kinase. LMP1-activated NF-κB signal pathway can promote the expression of downstream Pim1, thus promoting cell proliferation. Quercetagetin, an inhibitor of Pim1, can significantly inhibit the proliferation activity of NPC cells that express LMP1.^109^

In the view of the key role of inhibitor of NF-κB kinase (IKK) in the activation of the NF-κB pathway, IKK inhibitor is considered as one of the strategies of targeted therapy for cancer.^110^ The natural flavonoid glycoside vitexin can inhibit the activity of IKK thereby inhibiting the activation of the NF-κB signal pathway and inducing NPC cell apoptosis.^111^ BST2, also known as CD317 or HM1.24, is found to be highly expressed in cisplatin-resistant NPC cells. It can activate the NF-κB signal pathway and increase the expression of downstream anti-apoptotic proteins. BST2 overexpression is associated with a low survival rate in patients with NPC. Therefore, BST2 could be used as a prognostic marker and therapeutic target for NPC.^112^

Decreased expression of various components that inhibit the NF-κB pathway in NPC cells is one of the reasons for the abnormal activation of the NF-κB pathway. SIRT6, a kind of deacetylase, is downregulated in NPC cells. Lei et al. found that overexpression of SIRT6 might inhibit the expression of NF-κB by means other than acetylation and promoted apoptosis of NPC cells.^113^ In addition, knockout of NEAT1, a cancer-related long non-coding RNA, can inhibit the proliferation of NPC cells and promote apoptosis via promoting the activity of the miR-124/NF-κB axis.^114^ Deleted in liver cancer-1 (DLC-1) is a member of the GTPase-activating protein (GAP) family that is able to inhibit multiple tumor processes.^115^ Huang et al.^116^ demonstrated that DLC-1 could induce mitochondrial apoptosis and inhibit EMT and related processes by inhibiting the activation of the EGFR/Akt/NF-κB axis. Tumor necrosis factor-alpha-induced protein 3 (TNFAIP3), which has the function of the ubiquitin-editing enzyme, can inhibit the upstream signal transduction of the NF-κB signal pathway.^117^ Relative studies have shown that miR-19b-3p and miR-125b can directly suppress TNFAIP3 expression, thereby activating the NF-κB signal pathway, promote the proliferation of NPC cells, and inhibit the apoptosis of NPC cells.^118,119^ Ras-like estrogen-regulated growth inhibitor (RERG) is regarded as a potential tumor suppressor gene, expressed in a variety of normal tissues.^120,121^ Zhao et al. found that RERG was silenced by DNA hypermethylation in NPC cells. The extracellular-signal-regulated kinases (ERK)/NF-κB pathway is suppressed after RERG demethylation activation, which results in downregulating the expression of MMPs and pro-angiogenic cytokines, reducing the proliferation, migration, invasion, colony formation, and angiogenesis of NPC cells.^122^ Being a key active subunit of the NF-κB pathway, p65 is closely involved in the deregulation of the NF-κB pathway.^123^ Li et al.^124^ found that Epigallocatechin-3-gallate (EGCG) could inhibit NF-κB p65 activity, thus suppressing cancer progression, which is relevant to CSCs and EMT.

The NF-κB pathway can also be combined with immunotherapy. Huang et al.^125^ found that treating drug-resistant NPC cells with sunitinib could promote the expression of nature killer group 2 member D ligands (NKG2DLs) on the cell membrane by activating the NF-κB signal pathway, thereby enhancing NK cell-mediated cytotoxicity.

To sum up, the targeted inhibition of p65, LMP1, IKK, CDH6, Pim1, BST2, NEAT1, and the targeted activation of SIRT6, DLC-1, TNFAIP3, RERG, NKG2DLs in the targeted therapy of NPC could be a potential therapeutic strategy through the NF-κB pathway.

### MAPK pathway

MAPK is a member of the serine/threonine kinases family. There are three main subfamilies of MAPK: the ERK, the c-Jun N-terminal (JNK) or stress-activated protein kinases (SAPK), and MAPK14 (P38-α).^126^ The cascade activation of MAPK is an important pathway for the survival, proliferation, and drug resistance of cancer cells.^127^ In the MAPK pathway, recurrent mutations of its activators or regulators (FGFR2, FGFR3, BRAF1, NF1, and ERBB3) were detected in NPC samples.^25^ Hotspot mutations of KRAS, HRAS, and NRAS genes, promoter hypermethylation of RASAL, and DAB2 genes are also found.^29,128^

As an upstream kinase of the p38/MAPK pathway, the overexpression of mitogen-activated protein kinase kinase 6 (MAP2K6) is related to the radioresistance and poor prognosis of patients with NPC.^129^ In the MAPK/ERK pathway, PAK1 can phosphorylate Raf1 at Ser338 and MEK1 at Ser298, thereby activating the MAPK pathway.^130,131^ Franck et al. found that the macrocyclic lactone antibiotic ivermectin (IVM) could act as a PAK1 inhibitor to block the phosphorylation process of Raf1 and MEK1, resulting in cytotoxicity to NPC cells.^132^ After the MAPK pathway activation, it can phosphorylate and activate MAP kinase interacting serine-threonine kinase 1 (MNK1) which has been proved to be overexpressed in many kinds of cancers.^133,134^ Zhang et al.^135^ designed an MNK1 inhibitor compound 12dj that could inhibit the phosphorylation of downstream eukaryotic initiation factor 4E (eIF4E), thus producing cytotoxicity against NPC cells which is possibly related to the apoptotic cell death subroutine. BLU, a tumor suppressor gene, is found to disrupt cell cycle progression and result in the suppression of tumor growth, which is related to downregulated ERK signaling and the corresponding downstream effector cyclins D1 and B1.^136^

MiR-483-5p directly decreases death-associated protein kinase 1 (DAPK1) protein expression, increases colony formation of NPC cells, reduces radiation-induced apoptosis and DNA damage, via activating the ERK signal pathway.^137^ On the contrary, miR-124 can inhibit the occurrence of NPC through the MALAT1/ERK/MAPK axis.^138^ In addition, Peng et al.^139^ found that the suppression of proliferation, invasion, and metastasis of NPC by miR-124 is achieved by regulation of *Homo sapiens* forkhead box Q1 (Foxq1). Peptidyl-prolyl cis-trans isomerase NIMA-interacting 1 (PIN1), as an important signal regulator, is associated with EBV infection.^140^ Xu et al.^141^ found that PIN1 was overexpressed in EBV-associated NPC cells and PIN1 inhibitors Juglone could eliminate the PIN1 effect of activating the MAPK/JNK pathway, reducing the downstream protein cyclin D1 expression and enhancing the activity of caspase 3, finally inhibiting proliferation. PDZ binding kinase (PBK) is a kind of MAPK kinase (MAPKK) that is able to participate in many cellular functions through phosphorylating P38, JNK, and ERK.^142–144^ Wang et al.^145^ applied the PBK inhibitor HI-TOPK-032 to NPC cells and found that inhibiting PBK induced the oxidative stress by activating JNK/P38 signals, resulting in the accumulation of reactive oxygen species (ROS) and cell apoptosis.

To sum up, the targeted inhibition of MAP2K6, PAK1, MNK1, FGF2, PIN1, PBK, and the targeted activation of DAPK1, BLU, miR-124 in the targeted therapy of NPC could be a potential therapeutic strategy through the MAPK pathway.

### STAT3 pathway

Signal transducer and activator of transcription 3 (STAT3), a transcription factor encoded by the STAT3 gene, is a member of the STAT protein family.^146^ STAT3 is phosphorylated by Janus kinase (JAK) to have the role of activator of transcription and mediate a series of signal cascade reactions.

In vivo, miRNA is involved in the regulation of the STAT3 pathway. Recently a study showed that miR-29a can inhibit the 3′-UTR of STAT3 mRNA, reduce the level of STAT3, phosphorylated STAT3 and Bcl-2, inhibit cell proliferation, and enhance the level of apoptosis and drug sensitivity.^147^ Li et al.^148^ found that sulforaphane can upregulate the level of miR-124-3p in NPC cells, inhibit the 3′-UTR of STAT3 mRNA, enhance cell apoptosis, and inhibit cell proliferation. In addition, miR-124-3p was found to be upregulated in UCA1 gene knockout NPC cells and inhibition of the proliferation, invasion, and migration of NPC cells by miR-124-3p was achieved by inhibiting the expression of integrin β1 (ITGB1).^149^

EBV has previously been proposed to be particularly relevant to the onset of NPC. A quintessential example should be cited that is LMP1, which may contribute to the development of NPC through the STAT3 signal pathway. Ding et al.^109^ revealed that LMP1 can promote the activation of STAT3, protein kinase C (PKC), NF-κB, and AP1 in EBV-associated NPC cell NPC cells, thus activating Pim1 and promoting the proliferation of NPC cells.

He et al.^150^ showed that the downregulation of Raf kinase inhibitory protein (RKIP) can activate the signal transduction of the STAT3 pathway, enhance the migration and invasion ability of NPC cells in vitro, besides it can also enhance the metastasis and EMT in vivo. Furthermore, Huang et al.^151^ found that miR-181a can enhance radioresistance by inhibiting RKIP.

Eph receptors, a member of the receptor tyrosine kinase family (RTKs), participate in the regulation of the STAT3 signal pathway. Eph2 is often overexpressed in human malignant tumors, accompanied by the absence of Ephrin-A1, which can promote tumor invasion and metastasis, induce EMT, and maintain the cancer stem cell characteristics.^152^

JAK also has an essential role in the regulation of the STAT3 signal pathway. It is reported that IL-6 can activate lncRNA DANCR through the STAT3 pathway, forming a positive feedback loop, enhancing the invasion and proliferation ability of NPC cells in vitro.^153^ Liu et al.^154^ indicated that ovatodiolide could significantly inhibit the JAK2/STAT3 signal pathway, upregulate the level of Bax and Slug and decrease the level of Bcl-xL, c-Myc, and cyclin D1, which significantly reduces the cancer stem cell characteristics of NPC cells together with survival, proliferation, invasion, migration, and EMT inhibited, and promote the apoptosis of NPC cells.

For STAT3 pathways, except for directly targeting STAT3 for NPC treatment, targeted inhibition of LMP1, AP1, LncRNA DANCR, and targeted activation of miR-29a, miR-124-3p, and RKIP may become potential treatment strategies for NPC.

### Wnt/β-catenin pathway

Wnt/β-catenin signal pathway disorder is closely related to the occurrence and development of cancers.^155^ As a class of secreted glycoproteins, Wnt can bind to the receptors on the cell membrane and regulate the content of β-catenin in cells. β-catenin combines with T cell factor (TCF)/lymphoid enhancing factor (LEF) family in the nucleus, regulating the expression of the downstream genes.^156^ The promoter region of Wnt inhibitory factor 1 and SOX1 have been reported to be frequently hypermethylated in NPC which is in connection with the Wnt/β-catenin pathway activation.^157,158^

β-catenin is a key molecule in the Wnt/β-catenin signal pathway.^159^ Li et al.^160^ found that β-catenin could promote the expression of manganese superoxide dismutase (MnSOD), thereby reducing the level of ROS and increasing the resistance of anoikis in NPC cells. A small nuclear protein Chibby is able to inhibit β-catenin transcriptional activation through binding to its carboxyl terminus and decrease β-catenin content in the nucleus by cooperating with 14-3-3 adaptor proteins to transport it out of the nucleus, thereby inhibiting cell proliferation.^161^ MiR-34c directly suppresses β-catenin expression, thereby inhibiting proliferation, EMT, and radioresistance of NPC cells.^162^ Calpain small subunit 1 (Capn4), a small regulatory subunit of the Calpain family, has been shown to be highly expressed in a variety of cancers.^163–165^ MiR-124 can target Capn4 and then suppress the expression of β-catenin, cyclin D1, and c-Myc, leading to the inhibition of proliferation, invasion, and migration of NPC cells.^166^

Glycogen synthase kinase 3β (GSK3β), adenomatosis polyposis coli (APC), Axin and casein kinase Iα (CKIα) forms degradation complex to combine with β-catenin. GSK3β phosphorylates the Ser/Thr residues at the N-terminal of β-catenin, leading to the degradation of β-catenin.^167^ Huang et al.^168^ found that miR‑150 directly inhibited GSK3β expression at the protein level, resulting in the abnormal accumulation of β-catenin, leading to radiotherapy resistance of NPC cells. CREB binding protein (CBP), which co-activates and acetylates β-catenin with a promoter-specific pattern, is highly expressed in NPC cells.^169,170^ A small-molecule Wnt modulator ICG-001 can bind to CBP, thereby suppressing Wnt/β-catenin signal pathway and resulting in the reduction of the CSC population.^171^ MiR-506 could inhibit tumor growth and metastasis in NPC by downregulating LIM Homeobox 2 (LHX2), through the inactivation of the Wnt/β-catenin pathway.^172^ Furthermore, Forkhead box O3a (FOXO3a) silencing could induce radioresistance and EMT in NPC cells through activation of the Wnt/β-catenin pathway.^173^

To sum up, the targeted inhibition of β-catenin, Capn4, CBP, LHX2, FOXO3a, and the targeted activation of GSK3β in the targeted therapy of NPC could be a potential therapeutic strategy through the Wnt/β-catenin pathway.

### EGFR pathway

EGFR, also known as HER1 or ErbB1, is the first member that has been discovered in the ErbB family of tyrosine kinase receptor, which can bind with EGF, transforming growth factor-alpha (TGF-α), amphiregulin (AR), heparin-binding EGF-like growth factor (HB-EGF), and betacellulin (BTC); thus, signal cascade reaction and gene transcription are initiated in the cell. Downstream signal cascades of EGFR including RAS/RAF/MEK/MAPK/ERK, PI3K/Akt, PKC, Src, and JAK/STAT pathways, are crucial for cell proliferation, angiogenesis, apoptosis inhibition, cell motility, metastasis, adhesion, and gene expression.^174^

EGFR overexpression is common in NPC cells. Gu et al.^175^ found that the combination treatment of cetuximab and cisplatin for NPC cells can downregulate the levels of EGFR, phosphorylated EGFR, and phosphorylated Akt, upregulate the levels of Bax and caspase 3, and inhibit cell growth by increasing cisplatin-induced apoptosis, which indicated that the activation of EGFR/Akt pathway in NPC cells can inhibit the level of apoptosis, causing cisplatin resistance. In addition, serine protease inhibitor Kazal-type 6 (SPINK6) can promote cancer metastasis by binding and activating EGFR in NPC.^176^

In the treatment of NPC, targeted therapy for EGFR is still rarely approved for clinical use. It has been found that the progression-free survival (PFS) and overall survival (OS) of patients administered with nimotuzumab (NTZ) and intensity-modulated radiotherapy (IMRT) were significantly prolonged comparing with patients without adjuvant chemotherapy, and patients treated with cetuximab (CTX) and concurrent chemoradiotherapy (CCRT) had a significant improvement in OS, PFS and distant metastasis-free survival comparing with patients without cetuximab, but there is no significant difference in OS between anti-EGFR therapy using CTX/NTZ combined with palliative chemotherapy (PCT) and single PCT treatment.^177–180^

For EGFR pathways, apart from directly targeting EGFR for NPC treatment, targeting DLC-1, as well as a combination of EGFR targeting inhibitors with chemoradiotherapy may be potential therapeutic strategies for NPC.

### VEGF pathway

Notably, VEGF not only has an important role in leukemia and lymphoma but also is highly expressed in various solid malignant tumors, which is involved in the progress of malignant tumor diseases. The overexpression of VEGF in tumor tissue is closely related to the increase in angiogenesis, proliferation, and metastasis.^181^ Neuropilin-1 (NRP-1) is a VEGF receptor, which is co-expressed with VEGF in many tumor cells. It has been reported that NRP-1 can promote tumor growth.^182^ Using shRNA silencing NRP-1, Sun et al.^183^ discovered a vital inhibitory effect on the proliferation of CNE-2Z cells in vitro. Fu et al.^184^ found that lncRNA HOX transcript antisense intergenic RNA (Hotair) was significantly upregulated in NPC cells and clinical specimens, which can directly enhance the VEGF transcription activity, or indirectly enhance the VEGFA transcription activity and angiopoietin 2 (Ang2) activity through GRP78, mediating angiogenesis and tumor growth in NPC. Contrarily, miR-495 can directly target the 3′-UTR of GRP78, leading to the radiosensitivity of NPC.^185^

However, in the practical application, the clinical effect of conventional methods is limited as a result of the severe side effects. Li et al.^186^ studied the combination of bevacizumab and radiotherapy and chemotherapy and found that this combination treatment had a higher disease relief rate and a lower incidence of side effects, which can be used for further clinical experimental research.

### Endoplasmic reticulum stress

Endoplasmic reticulum (ER) is directly linked to the synthesis and folding of membrane and secretory proteins. When cells suffer from physiological stress and pathological stress, the balance between demand for protein folding and ER processing ability can be broken, thus ER stress occurs. In the occurrence of ER stress, a series of signal transduction pathways in cells will be activated, which are collectively referred to as unfolded protein response (UPR).^187^

ER stress is involved in the treatment of tumors. Different chemotherapy drugs can have different effects through the UPR signal pathway, as for etoposide, activation of the UPR signal pathway can increase the cell resistance,^188^ while it may enhance cisplatin sensitivity and induce apoptosis.^189^

It was also reported that the combination treatment of lenvatinib and iodine-131 increased the expression of ATF-6, IER1 RERK, CHOP, JNK, p38, and caspase 3 in NPC cells, indicating that the treatment-induced apoptosis of NPC cells by upregulating ER stress.^190^ Recently, Pan et al. found that the use of a curcumin compound (B63) can promote cell apoptosis, inhibit cell proliferation, and arrest the cell at the G2/M stage. The level of CHOP, ATF-4, and XBP-1 protein was significantly upregulated, suggesting that the activation of ER stress pathway may have a significant role in the anti-tumor effect of B63.^191^ Lin et al.^192^ revealed that Tetrandrine (TET) treatment of NPC cells increased apoptosis, upregulated the expression level of calpain 1, calpain 2, caspase 12, IRE-1α, IRE-1 β, GADD153, Glucose-regulated protein 78 (GRP78), ATF-6α, and ATF-6, indicating that TET induced cell apoptosis through ER stress.

**Fig. 2:**
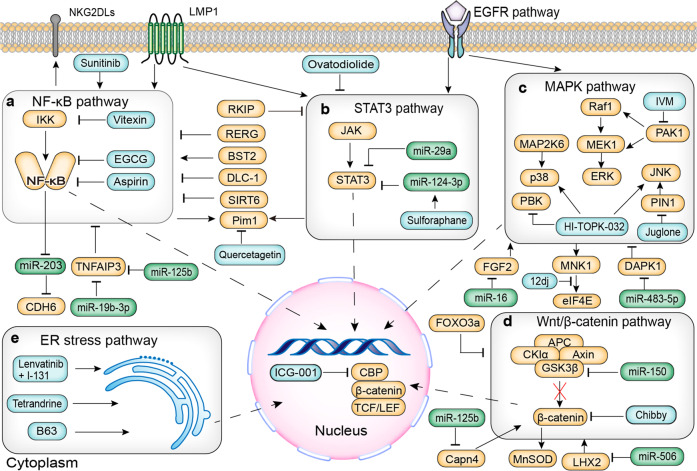
NF-κB pathway, MAPK pathway, STAT3 pathway, Wnt/β-catenin pathway, and ER stress pathway in NPC. **a** NF-κB pathway. **b** The STAT3 pathway. **c** MAPK pathway. **d** Wnt/β-catenin pathway. **e** ER stress pathway. The orange rounded rectangles indicate target molecules. The green rounded rectangles indicate microRNAs. The blue rounded rectangles indicate potential drugs. NF-κB nuclear factor kappa B, IKK inhibitor of NF-κB kinase, EGCG epigallocatechin-3-gallate, CDH6 cadherin 6, TNFAIP3 tumor necrosis factor-alpha-induced protein 3, RERG Ras-like estrogen-regulated growth inhibitor, DLC-1 deleted in liver cancer-1, Pim1 provirus integration site for Moloney murine leukemia virus 1, NKG2DLs nature killer group 2 member D ligands, STAT3 signal transducer and activator of transcription 3, JAK Janus kinase, RKIP Raf kinase inhibitory protein, EGFR epidermal growth factor receptor, LMP1 latent membrane protein 1, MAPK mitogen-activated protein kinase, PAK1 p21-activated kinase 1, IVM ivermectin, ERK extracellular-signal-regulated kinases, JNK c-Jun N-terminal kinases, MAP2K6 mitogen-activated protein kinase kinase 6, PBK PDZ binding kinase, PIN1 peptidyl-prolyl *cis*-*trans* isomerase NIMA-interacting 1, MNK1 MAP kinase interacting serine/threonine kinase 1, eIF4E eukaryotic initiation factor 4E, FGF2 fiber cell growth factor 2, DAPK1 death-associated protein kinase 1, TCF T cell factor, LEF lymphoid enhancing factor, CBP CREB binding protein, GSK3β glycogen synthase kinase 3β, APC adenomatosis polyposis coli, CKIα casein kinase Iα, MnSOD manganese superoxide dismutase, LHX2 LIM Homeobox 2, FOXO3a Forkhead box O3a, Capn4 Calpain small subunit 1, ER endoplasmic reticulum

Signal pathways in NPC, including the NF-κB pathway, MAPK pathway, STAT3 pathway, Wnt/β-catenin pathway and ER stress pathway can regulate their downstream genes’ expression, thereby influencing the biological behaviors of NPC cells. In each pathway, some potential targets and corresponding drugs mentioned in this review are shown in Fig. 2.

### Some other signal pathways related to the targeted therapy of NPC

Zhao et al.^193^ found that the Notch signal pathway may have a new function of conferring radioresistance on CNE-1 and CNE-2 cells, which is mediated by miR-20a-5p and neuronal PAS domain protein 2 (NPAS2). MiR-20a-5p can promote the invasion, metastasis, and radioresistance of NPC cells by inhibiting GTPases Rab27B, an important role in the development and metastasis of tumors.^194,195^ As a suppressor and promoter in various tumors, TGF-β not only has inhibitive roles in tumorigenesis but also induces EMT and tumor migration.^196^ The expression of E-cadherin, N-cadherin, twist, and snail can be regulated by the TGF-β pathway activated by annexin A2, which mediates the EMT process of NPC TW01 cells.^197^ Wang et al.^198^ found that berberine can reduce the activation of the TGF-β signal pathway induced by specificity protein 1 (Sp1) in CNE-2 cells, thus enhancing radiosensitivity. Nicotine negatively regulates miR-296-3p which directly targets the Ras/Braf/Erk/Mek/c-Myc pathway mediated by oncogenic protein MK2, promoting cytoplasmic transposition of c-Myc and the chemotherapy resistance, cell cycle progression, and EMT process of NPC.^68^ HIF-1 signal pathway is mainly regulated by the stability and activity of HIF-1α, and the overexpression of HIF-1α can lead to poor prognosis of NPC.^199^ Chen et al.^200^ constructed a kind of nanoparticle that can load HIF-1α siRNA, which has an inhibitive effect on tumor growth in CNE-2 tumor models. AMPK/mTOR/HIF-1 pathway can regulate the angiogenesis and glycolysis of NPC, and the microRNA-BART1-5P encoded by EBV acts on the α1 catalytic subunit of AMPK to regulate tumor metabolism and angiogenesis.^201^ The aberrant regulation of store-operated Ca^2+^ entry (SOCE) is highly associated with the process of NPC. The Ca^2+^ channel blocker skf96365 can inhibit the colony formation and increase the cell death rate by interfering with the Ca^2+^ signal induced by EGF, and induce the apoptosis and cell cycle arrest of G2/M and S-phase in CNE-2 and HONE-1 cells through caspase pathway.^202^ NaBu can promote SOCE in 5-8F and 6-10B cells to increase Ca^2+^ inflow and induce apoptosis in NPC cells.^203^ In addition, the overexpression of WW domain-containing oxidoreductase (WWOX) gene can also promote apoptosis by accumulating the cleavage of Caspase 3.^204^

## Non-coding RNA related to targeted therapy of NPC

Long non-coding RNA (lncRNA) and microRNA (miRNA) are highly associated with various tumors. In the past 5 years, the mechanism of lncRNA and miRNA in the progression, radioresistance, drug resistance, and angiogenesis of NPC has been studied.^184,205–207^ The role of lncRNAs and miRNAs in different NPC cell lines and the corresponding mechanisms have been, respectively, listed in Tables 1 and 2.

**Table 1:** Potential long non-coding RNA targets and related molecular mechanisms in NPC

LncRNA	Cell lines	Roles	Effects on NPC cells	Corresponding signal pathway	References
ANRIL	5-8F, CNE-1, CNE-2 and HONE-1	Oncogene	↑Proliferation ↓Apoptosis ↓Radiosensitivity	↑ANRIL/↓miR-125a	^205^
XIST	CNE-1, CNE-2	Oncogene	↑Proliferation ↓Radiosensitivity	↑XIST/↓miR-29c	^206^
NEAT1	HK-1, CNE-1, CNE-2 subclone S18 and SUNE-1 subclone 5-8F	Oncogene	↑Proliferation ↑Cisplatin resistance	↑NEAT1/↓let-7a-5p/↓RSF-1 Ras/MAPK signal pathway	^207^
Hotair	CNE-1, CNE-2	Oncogene	↑Proliferation ↑Angiogenesis	↑Hotair/↑VEGFA or ↑ GRP78/↑Ang2	^184^
CASC2	SUNE-1, SUNE-2, 6-10B	Tumor suppressor gene	↓Proliferation ↑Apoptosis	↓CASC2/↑miR-18a-5p/↓RBBP8 axis	^208^
CASC9	CNE-1	Oncogene	↑Proliferation ↑Glycolysis Biomarker of poor prognosis	HIF-1 signal pathway	^218^
FAM225A	CNE-1, CNE-2, HONE-1, SUNE-1, HNE-1, 5-8F, 6-10B, C666-1 and HK-1	Oncogene	↑Proliferation ↑Invasion	miR-590-3p, miR-1275/↑ITGB3/FAK/PI3K/Akt pathway	^70^
UCA1	5-8F, CNE-2	Oncogene	Tumor promotor ↑Proliferation ↑Invasion ↑Migration	↑UCA1/↓miR-145/↑ADAM 17	^219^
LINC00460	5-8F	Oncogene	↑Metastasis ↑Invasion ↑EMT	↑LINC00460/↓miR-30a-3p/↑Rap1A	^220^
ANCR	CNE-1, CNE-2, SUNE-1, C666-1, HONE-1 and HNE-1	Oncogene	↑Proliferation ↑Radioresistance	↑ANCR/↓PTEN	^226^
MALATA1	5-8F, CNE-2	Oncogene	↑Radioresistance ↑Activity of cancer stem cells	MALAT1/miR-1/slug axis	^227^
PVT1	5-8F, CNE-2	Oncogene	↑Radioresistance ↓Apoptosis Biomarker of poor prognosis	ATM–p53 pathway Caspase pathway	^228^

“↑” means upregulation, “↓” means downregulation, *EMT* epithelial–mesenchymal transition, *ANRIL* CDKN2B antisense RNA 1, *XIST* X inactive-specific transcript, *Rsf-1* remodeling and spacing factor 1, *VEGFA* vascular endothelial growth factor A, *GRP78* glucose-regulated protein 78, *Ang2* angiogenin 2, *CASC2* cancer susceptibility candidate 2, *RBBP8* retinoblastoma binding protein 8, *HIF-1* hypoxia induced factor 1, *ITGB3* integrin β3, *UCA1* urothelial carcinoma-associated 1, *Rap1A* Ras-related protein 1A, *ANCR*, antidifferentiation non-coding RNA, *PTEN* phosphatase and tensin homolog, *MALAT1* metastasis-associated lung adenocarcinoma transcript 1

**Table 2:** Functions and mechanism of miRNAs in NPC cells

MiRNA	Cell lines	Roles	Functions	Mechanism	References
miR-26b	CNE-2, HNE-1	Tumor suppressor gene	↑Cisplatin sensitivity	FOXD3/↑miR-26b /↓JAG1	^225^
miR-7	CNE-1, CNE-2	Tumor suppressor gene	↓Proliferation, ↓invasion	miR-7/↓Skp2/p57↑	^210^
miR-16	CNE-1, CNE-2	Tumor suppressor gene	↓Proliferation, ↓invasion, ↓migration	miR-16/↓FGF2/↓PI3K/Akt pathway	^77^
miR-1	5-8F, CNE-2	Tumor suppressor gene	↑Radiosensitivity	MALAT1/↓miR-1/↑slug	^227^
miR-124	6-10B	Tumor suppressor gene	↓Migration, ↓proliferation, ↓EMT	miR-124/↓MALAT1/ERK/MAPK axis; NEAT1/↓miR-124/ NF-κB axis; miR-124/↓Capn4/↓ cyclin D1 and c-Myc; miR-124/↓Foxq1	^138,139,114,167^
miR-148b	CNE-2, C666-1	Tumor suppressor gene	↓Proliferation, ↓migration, ↓invasion	miR-148b/↓MTA2	^314^
miR-149	6‐10B, 5‐8F	Oncogene	↑Proliferation, ↑invasion, ↑migration	miR-149/↓PKP3	^222^
miR-150	CNE-2, HONE-1	Tumor suppressor gene	↓Proliferation	miR-150/↓CDK2, CCND1, CCND2, CCNE2	^215^
miR-181a	CNE-2-IR, CNE-2	Oncogene	↑Radioresistance	miR-181a/↓RKIP	^151^
miR-212	6-10B, CNE-2	Tumor suppressor gene	↓Metastasis, ↓invasion	miR-212/↓SOX4	^211^
miR-324-3p	5-8F	Tumor suppressor gene	↓Proliferation, ↓Invasion, ↓metastasis	miR-324-3p /↓GLI3	^209^
miR-504	CNE-2-IR, HK-1-IR	Oncogene	↑Radioresistance	miR-504/↓NRF1	^231^
miR‑18a‑5p	NPC cells	Oncogene	↑Proliferation	↓CASC2/↑miR-18a-5p/↓RBBP8	^208^
miR‑495	5-8F, 5-8F-IR	Tumor suppressor gene	↑Radiosensitivity	miR-495/↓GRP78	^185^
miR-138-5p	HONE-1, HK-1	Tumor suppressor gene	↑Radiosensitivity	miR-138-5p /↓EIF4EBP1/ ↑eIF4E	^229^
miR-29a	CNE-2R, CNE-2,	Tumor suppressor gene	↑Radiosensitivity	miR-29a-3p /↓COL1A1	^79^
miR-20a-5p	CNE-1, CNE-2	Oncogene	↑Radioresistance, ↑migration, ↑invasion	miR-20a-5p /↓Rab27B	^195^
miR-29c	HNE-1, CNE-2	Tumor suppressor gene	↓Proliferation	miR-29c/↓HBP1/↓cyclin D1	^216^
miR-19b-3p	CNE-1, CNE-2	Oncogene	↑Radioresistance	miR-19b-3p /↓TNFAIP3/NF-κB axis	^118^
miR-142-3p	CNE-2, SUNE-1	Tumor suppressor gene	↓Metastasis	miR-142-3p /↓ZEB2	^63^
miR-124-3p	SUNE-1, C666-1	Tumor suppressor gene	↓Proliferation, ↓migration, ↓invasion	miR-124-3p /↓ITGB1	^149^
miR-152	CNE-2	Tumor suppressor gene	↓Invasion, ↓migration	miR-152/↓DNMT1	^62^
miR-101	CNE-2, 5-8F	Tumor suppressor gene	↓Metastasis, ↓invasion, ↓angiogenesis	miR-101/↓ITGA3	^214^
miR-483-5p	CNE-1, 5-8F	Oncogene	↑Radioresistance	miR-483-5p /↓DAPK1	^137^
miR-432	CNE-2, 5-8F	Tumor suppressor gene	↓Invasion, ↓metastasis	miR-432/↓E2F3	^212^
miR-185-3p, miR-324-3p	CNE-2	Tumor suppressor gene	↓Proliferation	miR-185-3p, miR-324-3p /↓SMAD7	^217^
miR-92a	5-8F, 6-10B	Oncogene	↑Metastasis, ↑invasion	miR-92a/↓PTEN/Akt pathway	^74^
miR-506	CNE-2, 5-8F	Tumor suppressor gene	↓Proliferation, ↓migration, ↓invasion	miR-506/↓LHX2/↓TCF4, ↓Wnt/β-catenin pathway	^172^
miR-378	5-8F, 6-10B	Oncogene	↑Proliferation, ↑migration, ↑invasion	miR-378/↓TOB2	^315^
miR-9	5-8F, CNE-1	Tumor suppressor gene	↓Angiogenesis	miR-9/↓MDK/↓PDK/Akt pathway	^72^

“↑” means promoting, “↓” means inhibiting, *JAG1* Jagged1, *Skp2* S-phase kinase-associated protein 2, *FGF2* fibroblast growth factor 2, *MALAT1* metastasis-associated with lung adenocarcinoma transcript 1, *MTA2* metastasis-associated gene 2, *PKP3* Plakophilin3, *CCND1* cyclin D1, *CCND2* cyclin D2, *CDK2* cyclin-dependent kinase 2, *CCNE2* cyclin E2, *RKIP* Raf kinase inhibitory protein, *SOX4* SRY-box transcription factor 4, *GLI3* *Homo sapiens* GLI family zinc-finger 3, *NRF1* nuclear respiratory factor 1, *RBBP8* RB binding protein 8, *GRP78* glucose-regulated protein 78, *EIF4EBP1* eukaryotic initiation factor 4E binding protein 1, *eIF4E* eukaryotic initiation factor 4E, *COL1A1* collagen type I alpha 1 chain, *Rab27B* member RAS oncogene family, *HBP1* HMG-box transcription factor 1, *TNFAIP3* TNF alpha-induced protein 3, *ZEB2* Zinc-finger E-box binding homeobox 2, *ITGB1* integrin beta-1, *DNMT1* DNA methyltransferase 1, *ITGA3* integrin subunit alpha 3, *DAPK1* death-associated protein kinase 1, *E2F3* E2F transcription factor 3, *SMAD7* SMAD family member 7, *PTEN* Phosphatase and tensin homolog, *LHX2* LIM Homeobox 2, *TCF4* transcription factor 4, *TOB2* transducer of ERBB2

### The anti-cancer effect

Recent studies have found that some microRNAs and lncRNAs in tumor tissues are downregulated and show negative regulation of pathological activities of NPC.

lncRNA cancer susceptibility candidate 2 (CASC2) could inhibit proliferation, induce apoptosis through inhibiting the activation of miR-18a-5p/RB binding protein 8 (RBBP8) axis.^208^

MiR-324-3p can inhibit the invasion of tumor cells through its suppression of Homo sapiens GLI family zinc-finger 3 (GLI3) gene expression.^209^ High expressions of miR-7 inhibit the proliferation and invasion of NPC cells by downregulating the expression of S-phase kinase-associated protein 2 (Skp2) which reduces the ubiquitination degradation of its targets such as p21, p57, and E-cadherin.^210^ MiR-212 directly targets and inhibits the expression of SOX4, a transcription factor at its downstream target, thus inhibiting the migration and invasion of NPC cells.^211^ In addition, miR-432 inhibits the expression of E2F transcription factor 3 (E2F3) by binding to the 3′-UTR of E2F3, leading the inhibition of the invasion and migration of NPC cells.^212^ MiR-148b inhibits proliferation, invasion, and metastasis of NPC cells by inhibiting metastasis-related gene 2 (MTA2).^213^

MiR-101 suppresses the level of integrin subunit alpha 3 (ITGA3), leading to inhibition of metastasis and angiogenesis of the tumor.^214^ MiR-150 was found to block cell cycle and inhibit the proliferation of NPC cells by inhibiting the expression of cyclin-related genes such as CCND1, cyclin D2 (CCND2), cyclin-dependent kinase 2 (CDK2), and cyclin E2 (CCNE2).^215^ Arresting cell at G1/S-phase and inhibiting the proliferation of NPC cells by miR-29c can be achieved by reduction of the transcription factor HMG-box transcription factor 1 (HBP1), causing the downregulation of cyclin D1 and cyclin D3.^216^

MiR-185-3p and miR-324-3p regulate the growth and apoptosis of NPC, which can be partially achieved by targeting the 3′-UTR of SMAD7.^217^

### Promotion of cancer

Conversely, some miRNAs and lncRNAs are upregulated in cancer tissues and facilitate pathological activities related to NPC, including inhibition of apoptosis, promotion of tumor cell proliferation, invasion, and metastasis.

LncRNA ANRIL, the expression of which is upregulated in NPC tissues compared to the normal nasopharyngeal epithelium, could promote proliferation and inhibit apoptosis in NPC via sponging miR-125a.^205^ As a member of the CASC family, lncRNA CASC9 could activate HIF-1α, thus facilitating the tumorigenesis and glycolysis of NPC cells.^218^ The expression of oncogene ADAM 17 could be increased after lncRNA urothelial carcinoma-associated 1 (UCA1) sponges miR-145 and UCA1 in NPC is regarded as a tumor promoter.^219^ LINC00460 is highly associated with the EMT in NPC cells via binding miR-30a-3p to regulate the expression of Ras-related protein 1A (Rap1A).^220^ What’s more, patients whose infiltrating lymphocytes are characterized by high expression of lncRNA AFAP1-AS1 incline to distant metastasis and poor prognosis, especially with positive PD1.^221^ *N*,*N*′‐dinitrosopiperazine (DNP) can promote proliferation, invasion, and metastasis of NPC by enhancing the expression of miR-149 which suppresses the level of PKP3.^222^ It is mostly believed that RBBP8 is involved in the repair of DNA double-strand breaks in cancer.^223^

### Drug resistance

Combined chemotherapy is one of the main methods to treat patients with NPC, but in recent years, it has been found that the therapeutic effects of anti-cancer drugs are not satisfactory, the main reason for which is that NPC cells have developed resistance to anti-cancer drugs.

Triptonide, as a molecule monomer extracted from Chinese herbal medicine, can interfere with the lnc-THOR-IGF2BP1 signal and inhibit the proliferation of NPC cells in vitro and in vivo.^224^ Forkhead box protein D3 (FOXD3) can promote the expression of miR-26b, which inhibits the JAG1/Notch axis, inducing the sensitivity of NPC cells to cisplatin (CDDP).^225^

### Radioresistance

**Fig. 3:**
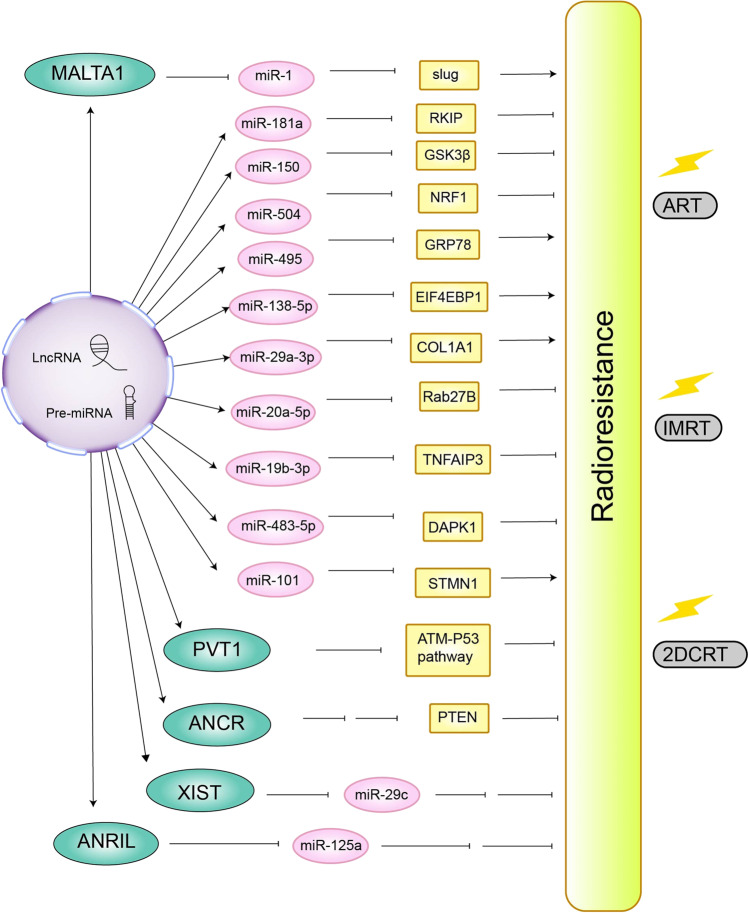
Mechanism of non-coding RNA on radioresistance of NPC. JAG1 Jagged1, Skp2 S-phase kinase-associated protein 2, FGF2 fibroblast growth factor 2, MALAT1 metastasis-associated with lung adenocarcinoma transcript 1, MTA2 metastasis-associated gene 2, PKP3 Plakophilin3, CCND1 cyclin D1, CCND2 cyclin D2, CDK2 cyclin-dependent kinase 2, CCNE2 cyclin E2, RKIP Raf kinase inhibitory protein, SOX4 SRY-box transcription factor 4, GLI3 *Homo sapiens* GLI family zinc-finger 3, NRF1 nuclear respiratory factor 1, RBBP8 RB binding protein 8, GRP78 glucose-regulated protein 78, EIF4EBP1 eukaryotic initiation factor 4E binding protein 1, COL1A1 collagen type I alpha 1 chain, Rab27B member RAS oncogene family, HBP1 HMG-box transcription factor 1, TNFAIP3 TNF alpha-induced protein 3, ZEB2 zinc-finger E-box binding homeobox 2, ITGB1 integrin β1, DNMT1 DNA methyltransferase 1, ITGA3 integrin subunit alpha 3, DAPK1 death-associated protein kinase 1, E2F3 E2F transcription factor 3, SMAD7 SMAD family member 7, PTEN phosphatase and tensin homolog, ANRIL CDKN2B antisense RNA 1, XIST X inactive-specific transcript, ANCR antidifferentiation non-coding RNA, 2DCRT two-dimensional radiotherapy, IMRT intensity-modulated radiotherapy, ART adaptive radiotherapy

The radioresistance of NPC is one of the main reasons for the low efficacy of radiotherapy. It is widely believed that some miRNAs are upregulated or downregulated in radiation-resistant NPC cells and can reduce or enhance the sensitivity of tumor cells to radiation. How non-coding RNAs induce or inhibit the radioresistance of NPC has been shown in Fig. 3.

The knockdown of lncRNA X inactive-specific transcript (XIST) could upregulate miR-29c, resulting in the inhibition of DNA damage repair and an increase of the radiosensitivity of NPC cells.^206^ Antidifferentiation non-coding RNA (ANCR) could regulate PTEN expression epigenetically to promote radioresistance.^226^ Besides, lncRNA MALAT1 modulates cancer stem cell activity of NPC and induces radioresistance via regulating the miR-1/slug axis.^227^ LncRNA NEAT1 downregulates remodeling and spacing factor 1 (Rsf-1) expression to activate the Ras-MAPK pathway to mediate NPC resistance to cisplatin through direct interaction with let-7a-5p.^114,207^ Downregulated expression of lncRNA PVT1 can also induce apoptosis of NPC cells through the DNA damage repair pathway after radiotherapy, thus being regarded as a prognostic indicator.^228^

By reducing the level of eukaryotic initiation factor 4E binding protein 1 (EIF4EBP1), miR-138-5p increases eIF4E, enhancing the autophagy of NPC cells induced by radiotherapy.^229^ MiR-101 enhances radiation-induced autophagy and radiation sensitivity of tumor cells by targeting Stathmin 1 (STMN1).^230^ By inhibiting nuclear respiratory factor 1 (NRF1), miR-504 interferes with mitochondrial-mediated oxidation reaction and enhances radioresistance.^231^

## Clinical significance

### Clinical trials

Failure of NPC treatment mainly includes distant metastasis, recurrence, radiation resistance, and drug tolerance. Targeted therapy blocks signal transmission by targeting specific molecules related to tumor progression in and out of NPC cells, thus inhibiting the occurrence and development of NPC. At present, most of the clinical trials of targeted therapy against NPC are clinical phase I or II trials, which show that different targeted drugs can delay the process of NPC to different degrees and prolong the life of patients.

At present, clinical trials on targeted therapy for NPC are not abundant, which are mainly targeting EGFR and VEGF/VEGFR. Drugs that target EGFR include gefitinib, nimotuzumab, cetuximab, h-R3, and so on while drugs targeting VEGF/VEGFR mainly include axitinib, aflibercept, bevacizumab, sorafenib, sunitinib.

In phase II clinical trial, gefitinib is well-tolerated by subjects along with a poor response rate, making it less suitable for clinical treatment.^232^ In contrast, nimotuzumab in combination with 5-fluorouracil is significantly effective and well-tolerated by subjects in the treatment of recurrent and metastatic NPC.^233^ In stage N3 NPC, induction chemotherapy and sequential nimotuzumab plus CCRT achieved a positive survival benefit with tolerable toxicity.^234^ Compared with helical tomotherapy (HT) combined with cetuximab followed by adjuvant chemotherapy (ACT) with docetaxel, HT combined with cetuximab followed by ACT with cisplatin is an effective treatment for locally advanced disease (LANC), which has a high survival rate and few side effects.^235^ In a phase II trial, cetuximab-radiotherapy has better therapeutic effects but shows more acute adverse effects than cisplatin-chemoradiotherapy.^236^ h-R3 can enhance the radiosensitivity of locally advanced NPC with good safety, but the long-term efficacy is not effective.^237^

In patients with NPC, the use of bevacizumab can show a better effect compared with corticosteroids.^238^ Adding bevacizumab to chemoradiation is an effective and feasible treatment, suggesting that bevacizumab may delay the progression of subclinical disease.^239^ Axitinib has good control of disease progression and safety in patients with severe pre-treated NPC.^240^ Aflibercept plus docetaxel has achieved preliminary efficacy in Chinese patients with NPC, and this treatment deserves further trials.^241^

Mk-2206 is an inhibitor of Akt, which has limited activity in the heavily pre-treated group of patients in a multicenter phase II clinical trial.^242^ Further studies are needed to select appropriate Akt inhibitors to treat NPC.

Famitinib, a tyrosine kinase inhibitor whose targets include VEGFR, platelet-derived growth factor receptor (PDGFR), and stem cell factor receptor (SCFR), has an encouraging anti-cancer profile and tolerability for patients with NPC along with chemoradiation. And it is necessary to expand the sample size to further confirm the efficacy.^243^ In NPC patients receiving high-dose radiation therapy, the incidence of upper respiratory tract bleeding will become higher after the use of sunitinib.^244^ Sorafenib has a certain anti-tumor effect and is well-tolerated by the subjects.^245^ The outcome of the phase II clinical trial has verified sorafenib combined with cisplatin and 5-FU is feasible and tolerable in patients with recurrent or metastatic NPC.^246^

Combination of endostar with gemcitabine–cisplatin chemotherapy can have good control of cancer progression and improve the prognosis of patients with NPC.^247^ Besides, endostar combined with IMRT has lower acute toxicity along with similar effects compared with IMRT.^248^

In clinical trials, the effect of targeted drugs alone is not good, whose PFS is generally less than one year, while targeted drugs combined with chemotherapy or radiotherapy can significantly delay the progress of the tumor, with a great improvement of PFS and OS.

In addition, there are some problems when studying clinical trials in targeted therapy of NPC:

1. The quantity of patients is small, which may cause the inaccuracy of the results.
2. Almost all the targets that occurred in clinical trials of NPC are VEGFR or EGFR and other new drugs targeting miRNAs or other key molecules can be developed.
3. More phase III clinical trials are needed after the drug has passed phase I and phase II clinical trials.

We summarize the current clinical trials related to targeted therapy for NPC, which are presented in Table 3.

**Table 3:** Present clinical trials about targeted therapy in NPC

Drug	Mechanism/target	Trial phase	Treatment schedule	Patients number	Patients characteristics	PFS	OS	References
Gefitinib	EGFR	II	Single drug	19	Recurrent metastatic NPC	–	16 months	^232^
Nimotuzumab	EGFR	II	Combined with cisplatin and 5-fluorouracil	39	Recurrent metastatic NPC	7.0 months	16.3 months	^233^
Nimotuzumab	EGFR	II	Induction chemotherapy, sequential Nimotuzumab plus concurrent chemoradiotherapy.	45	N3M0	79.5% (3 years)	85.6 (3 years)	^234^
Cetuximab	EGFR	II	Concurrent HT with cetuximab, followed by chemotherapy (docetaxel and cisplatin)	43	33 Stage III and 10 Stage IV	79.1% (2 years), 72% (3 years)	93.0% (2 years), 85.7% (3 years)	^235^
Cetuximab	EGFR	II	Cetuximab-radiotherapy	21	Stage III–IVb	95.2% (3 years)	100% (3 years)	^236^
h-R3	EGFR	II	Radiotherapy combined with h-R3	35	Stage III–IVb	–	–	^237^
Bevacizumab	VEGF	II		44	–	–	90.9% (2 years)	^238^
Bevacizumab	VEGF	II	Bevacizumab, corticosteroid-controlled	112	–	–	–	^239^
Axitinib	VEGFR	II	Single drug	40	Recurrent or metastatic NPC	5.0 months	10.4 months	^240^
Aflibercept	VEGF	I	Aflibercept plus docetaxel	16	–	–	–	^241^
Mk-2206	Akt	II	Single drug	21	Recurrent or metastatic NPC	3.5 months	10 months	^242^
Famitinib	VEGFR, PDGFR (platelet-derived growth factor receptor), SCFR (stem cell factor receptor)	I	Single drug	20	Stage III or IVa-b NPC	75% (3 years)	–	^243^
Sunitinib	multi-kinase inhibitor	II	Single drug	14	Previously received high-dose radiation	3.5 months	10.5 months	^244^
Sorafenib	multi-kinase inhibitor	II	Single drug	28	–	3.9% (6 months)	4.2 months	^245^
Sorafenib	multi-kinase inhibitor	II	Single drug	54	Recurrent or metastatic NPC	7.2 months	11.8 months	^246^
Endostar	VEGF, VEGFR, PDGFR	II	Combination of gemcitabine	30	–	19.4 months	90.2% (1 year)	^247^
Endostar	VEGF, VEGFR, PDGFR	–	Intensity-modulated radiotherapy combined with endostar	23	Stage III-IVa	100% (2 years)	100% (2 years)	^248^

“–” means not clear, *PFS* progression-free survival, *OS* overall survival

### Biomarkers and preclinical researches

PKB, mTOR, and PI3K in NPC tissues are highly upregulated and have higher positive rates than in normal nasopharyngeal tissues; Cox regression analysis reveals expressions of PI3K, PKB and mTOR are the major risk factors for the prognosis of NPC.^249^ High expression of LMP1 is strongly associated with NPC patients’ survival, which indicates LMP1 may be potential prognostic biomarkers.^250,251^ In a study of NPC epithelial tissue, high expression of HIF-1α is found to be a new independent biomarker for predicting poor prognosis in NPC patients.^252^ It is found that the serum BMI-1 antibody in patients is significantly higher than in normal persons, suggesting that BMI-1 antibody may be a potential biomarker of NPC and has diagnostic and prognostic value.^253^ CDH6 is overexpressed in the LMP1-positive NPC tissues, which can promote EMT and metastasis of NPC, which may be a therapeutic target of NPC.^107^ Multivariable Cox regression analysis shows that BST2 expression can serve as independent prognostic factors and high BST2 expression can predict poor prognosis in patients with locally advanced NPC treated with platinum-based chemoradiotherapy.^254^ The expression of NRP-1 is highly upregulated in the NPC tissues and the multivariate analysis indicates that the overexpression of NRP-1 is an independent prognostic factor for NPC patients.^255^ Multivariate Cox regression indicates that the elevated MAP2K6 is independently associated with poor prognosis in NPC patients.^129^ Phosphorylated MNK1 has a significantly higher expression in NPC tissues compared to the nasopharyngeal epithelium and may be independent prognostic factors of NPC.^256^ The concentration of DAPK1 methylation in serum is significantly higher compared with the normal group and may become a diagnostic biomarker for early NPC.^257^

Clinical analysis shows that the high expression of miR-92a is associated with adverse clinicopathological features such as lymph node metastasis and distant metastasis in advanced tumors, and miR-92a is a good biomarker for predicting the prognosis of NPC patients.^74^ Similarly, clinical parameter researches reveal that elevated miR-18a levels are associated with advanced stages of NPC, lymph node metastasis, human herpesvirus IV infection, and high NPC mortality.^258^ MiR-124-3p significantly elevates after treatment and decreases at recurrence or metastasis compared with pretreatment, which reveals miR-124-3p may serve as the prognostic biomarker in NPC.^259^ The expression of miR-185-3p and miR-324-3p is significantly decreased after RT in radioresistant, and combined low miR-185-3p and miR-324-3p may be vital markers for prediction of low response to RT/CRT.^217^ Expression of lncRNA Hotair in NPC tissues is higher than in nasopharyngeal tissues and the multivariate analysis reveals that Hotair is a potential biomarker for prognosis of NPC.^260^ LncRNA ANRIL is overexpressed in NPC tissues, which is related to the clinical stage and can serve as an independent predictor for NPC patients.^261^

In addition, Annexin A1, CDKN3, OCT4, c-Src, COX-2, TNFAIP3, RERG, PBK, STAT3, DANCR, miR-29a, β-catenin, c-Myc, and Capn4 can be used as prognostic markers in NPC. Annexin A1 is a potential biomarker for predicting response to RT of NPC and predicting NPC differentiation and prognosis.^262,263^ High expression of CDKN3 is an independent negative prognostic factor in NPC.^264^ OCT4 is significantly associated with EMT and can be used as an independent prognostic factor in NPC.^265^ Elevated serum c-Src levels are associated with poor prognosis in NPC patients.^86^ In NPC patients, aberrant expression of COX-2 is associated with recurrence and poor prognosis.^100^ Downregulation of TNFAIP3 is associated with distant metastasis and worse patient prognosis.^266^ The methylation rates of RERG can serve as novel biomarkers for early detection and screening of NPC.^267^ High-level PBK in NPC is significantly associated with poor prognosis.^145^ STAT3 is associated with a relatively good prognosis in NPC patients.^268^ LncRNA DANCR can be used as a biomarker for poor prognosis.^269^ MiR-29a can promote metastasis and invasion of NPC cells, and it would be an ideal prognostic marker.^270^ Positive expression of β-catenin and c-Myc is negatively correlated with the survival rate of NPC patients, demonstrating they can be used as important prognostic biomarkers.^271^ Capn4 can promote invasion and metastasis of NPC, which suggests that Capn4 may be an independent prognostic factor in NPC.^272^

The lack of preclinical models is an important reason that hinders the development of targeted therapies for NPC, and we summarize some meaningful preclinical studies.

PBK can promote the growth of NPC in nude mice and HI-TOPK-032, the specific inhibitor for PBK/TOPK, can significantly reduce the volume and weight of NPC in nude mice without obvious signs of toxicity.^145^ In Wong et al.’s^273^ research, the tumor weight of mice treated with the PI3K-mTOR dual inhibitor PF-0469150210 is significantly reduced when compared with the control group. Besides, the PF-04691502 has minimal effect on the animal’s body weight with no gross toxicity observed throughout the treatment.

Brevilin A can inhibit PI3K/Akt/mTOR and STAT3 signaling pathways in vitro and Brevilin A treatment led to no significant weight loss in treated mice. These contribute to the preclinical development of Brevilin A as a chemotherapeutic for NPC.^274^ Evofosfamide is a hypoxia-activated prodrug that selectively targets hypoxic regions in solid tumors. Since HIF-1α is overexpressed in NPC tissues, the results provide preclinical evidence that Evofosfamide is used as a single drug in combination with DDP to target the selective anoxic fraction of NPC.^275^ COX-2 can promote the occurrence and recurrence of NPC, and the cellular senescence of fibroblasts in COX-2 knockout mice is significantly increased, suggesting that COX-2 may be a potential indicator to predict NPC recurrence and treatment resistance as well as a target for targeted therapy of NPC.^100^ The expression of EGFR correlates with β-catenin in NPC patient specimens, and β-catenin is responsible for regulating CSC characteristics of EGFR/Akt activation. The results suggest that targeting β-catenin is a reasonable clinical treatment for NPC with high EGFR or Akt expression.^276^

## Discussion

The models for NPC research can be divided into two categories: in vitro models and in vivo models. The former includes the previous two-dimensional models and the current potential of the three-dimensional (3D) models.^277^ Two-dimensional models are effortless to build and maintained at a low cost.^278^ However, the lack of crosstalk with fibroblasts, immune cells and endothelial cells indicates that the tumor microenvironment in vivo cannot be entirely simulated, which is not convenient for drug penetration and drug resistance evaluation.^277,279,280^ The construction of 3D tumor spheroid models for NPC could be accomplished by EB virus-positive C666-1 cells, and also EB virus negative CNE-1 and CNE-2, HONE-1, and SUNE-1.^124,171,273,281^ Researchers can utilize different matrix to reconstruct tumor microenvironment, which guarantees drug sensitivity, the alterations of signaling pathways and gene expression in tumor cells.^277^ Benefiting from its simulation of body condition, they ensure the reliability and validity of the experimental results of the targeted drug candidates. However, the study used lapatinib, a tyrosine kinase inhibitor against EGFR, on HONE-1 spheroids exhibited that the drug did not diffuse effectively to core cells of mass.^282^ Organoids are a group of cells that undergo organ differentiation and directional self-organization of adult stem cells or pluripotent embryonic stem cells in vitro.^283^ The organoids from patients are called patient-derived organoids (PDO) models, which have the characteristics of high simulation, short culture period, and stable passage.^284^ It is considered as the most ideal preclinical model at present. Tumor organoids are obtained by using tumor cells harvested from patients to culture masses in vitro, which retains the genetic background and reproduces the microenvironment. PDO models are promising substitutes for patients, which can simulate the treatment response from the aspects of pathology, gene, cell and tumor microenvironment.^285^ Organoids can also be used as the model of NPC stem cells. The increased expression of tumor stem cell markers in organoids can be considered as the concentration and enrichment of tumor stem cells so that organoids behave similarly to patients on tumor recurrence and treatment resistance. Organoids have advantages in the study of intercellular interaction and immune microenvironment for it can stably carry the EB virus for a long time and mimic intercellular communication involving in exosomes.^286^ The test results of the breast cancer organoids for the HER-2 targeted drug gefitinib were consistent with the patients’ conditions, indicating that the organ model had similar properties to cancer in vivo.^287^

The construction of in vivo models could be divided into spontaneous models, induced models, transplantation models, and transgenic models.^288–292^ Spontaneous models are rarely obtained and utilized.^288^ Induction conforms to the characteristics of tumor dynamics and is often used to screen carcinogens but seldom used in targeted therapy for NPC.^289^ Transplantation models include subcutaneous xenotransplantation, orthotopic xenotransplantation, and patient-derived tumor xenografts (PDX).^293,294^ The first two tumors have different injection sites and in terms of the xenograft models, the tumor was in a mouse microenvironment rather than a human one. PDX models maintain the heterogeneity of tumor tissues, thus enabling phase I efficacy evaluation results to be more than 87% similar to clinical results.^295,296^ Several NPC PDXs are available for research in the past, including C18, C17, and C15.^297^ However, they passaged so long in nude mice that they may have altered the biological characteristics of original tumors. Thus, novel NPC PDXs models need to be established, normalized and unified to ensure the reliability of experimental results. Hsu et al.^298^ constructed a novel kind of PDX model to study the effect of anti-cancer drugs and treated a patient with liver metastasis of NPC with the same drugs and they confirmed that Palbociclib, a cyclin-dependent kinase inhibitor, had an anti-cancer effect, which helps to show the reliability of the application of PDX models in targeted therapy.

The products of EBV, lncRNAs, and miRNAs formulate a model of the regulatory network of metastasis in NPC.^299^ However, CSCs and EMT are closely related to recurrence and early metastasis in NPC. In NPC cells, some stem cell markers such as ALDH1, CD44, CD133, and Bim-1 have been confirmed. CSCs have biological characteristics including high self-renewal ability, pluripotent marker expression, and resistance to drug and radiation. Some signal pathways are important for the self-renewal and maintenance of stem cells.^300^ Nowadays, EMT activation is thought to be associated with the generation of CSCs.^301^ After activation of EMT, tumor epithelial cells transform into mesenchymal cells, with the loss of cell polarity and cell–cell adhesion and the gain of migratory and invasive property.^302^ Recent study showed that different mechanisms of EMT participate in cancer metastasis which includes cytoskeletal reorganization, altered expression of cell adhesion molecules, degradation of the basement membrane, and continuous autocrine growth factor signaling to evade apoptosis or anoikis.^303^ Serglycin has a vital role in enhancing NPC metastasis via enhancing EMT, cellular migration, and cellular invasiveness.^304^ Besides, IL-6 is able to reduce cell adherence toward laminin and stimulates the expression of MMP-2 and MMP-9, leading to metastasis of NPC.^305–307^ It is hoped that the emergence of targeted therapies against CSCs and EMT might bring about clinical benefits. However, continued study is still imperative in order to attain the goal of understanding the molecular expression profile together with the characteristics of signal pathways in CSCs.

The In-depth researches on targeted therapy of NPC are conducive to the clinical treatment of NPC. The PI3K/Akt pathway, which has an important role in NPC, can be activated by the EGF signal, and there are currently targeted drugs such as Cetuximab that target EGFR.^236^ Inhibitors of components in the PI3K/Akt pathway have been used to treat other cancers, and further studies are needed to determine whether they are appropriate for the treatment of NPC. P53 is an important tumor suppressor gene. There have been many studies on targeted therapy of p53. The main therapeutic methods are to restore the function of p53 and to interfere with the p53–MDM2 axis.^308^ By reviewing recent studies on PI3K/Akt pathway and p53 pathway in NPC, we have summarized some potential targets that can affect the PI3K/Akt pathway and p53 pathway, which will be conducive to the selection of targeted therapeutic targets for NPC in future basic studies. Meanwhile, the targeted drugs used to treat NPC in clinical trials mainly consist of EGFR inhibitor (Cetuximab, Nimotuzumab), VEGF/VEGFR inhibitor (Bevacizumab, Endostar). Most targeted drugs are still in the level of basic experiments, their clear mechanisms of action need a further investigation. However, compared with traditional treatment, the molecular targeted therapy brings less side effects, which can improve the prognosis of patients and increase the tolerance of treatment. Owing to the fact that the Notch signal pathway could be involved in the radioresistance of NPC, whether it has other roles in the progression and prognosis of NPC should be further studied. Radioresistance is the main cause of poor prognosis of advanced-stage NPC, thus molecular targets that reduce radioresistance and increase radiosensitivity should be thoroughly studied, and related drugs should be combined with radiotherapy to test efficacy and safety.

On the one hand, lncRNAs could modulate the related miRNA and downstream genes axis so that they have dual roles in NPC. On the other hand, the research and development of drugs targeting lncRNAs for the treatment of NPC are still rare and studies will be needed to develop new strategies based on lncRNAs in the future. MiRNAs have recently been found to be highly or lowly expressed in tumor cells, and some miRNAs have been shown to promote or inhibit the processes of NPC cells in vitro and in vivo. Future NPC treatment can inhibit the proliferation, metastasis, EMT or angiogenesis of tumor cells by inhibiting the relevant miRNAs. Some miRNAs can increase the sensitivity of tumor cells to anti-cancer drugs and can be used in combination with targeted therapeutic drugs to improve the efficacy, while other miRNAs can reduce the radioresistance of NPC cells and can be considered in combination with local IMRT. However, to carry out the above possible therapeutic methods, it is also necessary to continuously study the functions of molecules including proteins, DNA, and RNA on the cytoplasm and membrane of tumor cells and normal cells, gradually complete the relationship network in the tumor and perform cellular and animal experiments on potential targets. In addition, miRNAs have an important role in the maintenance of the tumor microenvironment and communication between tumor cells, and it can be explored to understand what the specific mechanism of miRNA involvement is, which in turn provides a theoretical basis for possible targeted therapy.

In addition to further study in NPC treatment, effective population screening may improve the detection rate of early-stage NPC, which is conducive to the early treatment of NPC. By now, the detection of early-stage NPC is mainly through EBA IgA antibody (EA-IgA), anti-EBV capsid antigen (VCA-IgA), anti-EBV nuclear antigen 1 (EBNA1-IgA), while the extensive application is restricted by the low sensitivity and specificity.^309^ Encouragingly, recent studies have found that plasma EBV DNA detection displays a better outcome on early-stage NPC detection.^310,311^ Besides, with the profound study of vaccine technology and the growing understanding of EBV immunology, an increasing number of vaccine candidates against EBV have been developed.^312,313^ Clearly, thorough studies are needed to provide more insight into early-stage NPC screening and NPC vaccines, which have a huge application foreground in the prevention and treatment of NPC in the future.
