# Correction: Advances in targeted therapy mainly based on signal pathways for nasopharyngeal carcinoma

**DOI:** 10.1038/s41392-020-00394-2

**Published:** 2020-11-13

**Authors:** Yuanbo Kang, Weihan He, Caiping Ren, Jincheng Qiao, Qiuyong Guo, Jingyu Hu, Hongjuan Xu, Xingjun Jiang, Lei Wang

**Affiliations:** 1 Department of Neurosurgery, Cancer Research Institute, Xiangya Hospital, Central South University, 410008 Changsha, Hunan China; 2 Cancer Research Institute, Collaborative Innovation Center for Cancer Medicine, School of Basic Medical Science, Central South University, 410008 Changsha, Hunan China; 3 The NHC Key Laboratory of Carcinogenesis and The Key Laboratory of Carcinogenesis and Cancer Invasion of the Chinese Ministry of Education, Xiangya Hospital, Central South University, 410008 Changsha, Hunan China

**Keywords:** Cancer, Oncology

Correction to: *Signal Transduction and Targeted Therapy* 10.1038/s41392-020-00340-2, published online 23 October 2020

Since the acceptance and publication of this article^1^, the authors opted to change the order of authors in the author group. The correct order of authors is: Yuanbo Kang, Weihan He, Caiping Ren, Jincheng Qiao, Qiuyong Guo, Caiping Ren, Jingyu Hu, Hongjuan Xu, Xingjun Jiang^1^ and Lei Wang

This has been corrected in the original version.
